# Dual PI3K/mTOR Inhibitor NVP-BEZ235 Enhances Radiosensitivity of Head and Neck Squamous Cell Carcinoma (HNSCC) Cell Lines Due to Suppressed Double-Strand Break (DSB) Repair by Non-Homologous End Joining

**DOI:** 10.3390/cancers12020467

**Published:** 2020-02-18

**Authors:** Ulrike Schötz, Viola Balzer, Friedrich-Wilhelm Brandt, Frank Ziemann, Florentine S.B. Subtil, Thorsten Rieckmann, Sabrina Köcher, Rita Engenhart-Cabillic, Ekkehard Dikomey, Andrea Wittig, Andrea Arenz

**Affiliations:** 1 Department of Radiotherapy and Radiooncology, Philipps-University, University Hospital Giessen and Marburg, 35043 Marburg, Germany; viola-balzer@t-online.de (V.B.); friedrich-wilhelm.brandt@uni-marburg.de (F.-W.B.); ziemann5@staff.uni-marburg.de (F.Z.); florentine.subtil@staff.uni-marburg.de (F.S.B.S.); Rita.Engenhart-Cabillic@uk-gm.de (R.E.-C.); dikomey@uke.de (E.D.); Andrea.Wittig@med.uni-jena.de (A.W.); arenza@staff.uni-marburg.de (A.A.); 2 Laboratory of Radiobiology & Experimental Radiooncology, University Medical Center Hamburg Eppendorf, 20251 Hamburg, Germany; t.rieckmann@uke.de (T.R.); s.koecher@uke.de (S.K.); 3 Department of Otorhinolaryngology and Head and Neck Surgery, University Medical Center Hamburg Eppendorf, 20251 Hamburg, Germany; 4 Department of Radiotherapy and Radiation Oncology, University Hospital-Jena, 07740 Jena, Germany

**Keywords:** radiation, radiosensitizer, radioresistance, HNSCC, Akt, DNA-PKcs, BEZ235

## Abstract

The PI3K/Akt/mTOR pathway is frequently altered in human papillomavirus (HPV)-positive and negative squamous cell carcinoma of the head and neck (HNSCC) and overstimulation is associated with poor prognosis. PI3K drives Akt activation and constitutive signaling acts pro-proliferative, supports cell survival, DNA repair, and contributes to radioresistance. Since the small molecule NVP-BEZ235 (BEZ235) is a potent dual inhibitor of this pathway, we were interested whether BEZ235 could be an efficient radiosensitizer. The 50 nM BEZ235 was found to abrogate endogenous and irradiation-induced phosphorylation of Akt (Ser473). The anti-proliferative capacity of the drug resulted in an increase in G1-phase cells. Repair of radiation-induced DNA double-strand breaks (DSBs) was strongly suppressed. Reduction in DSB repair was only apparent in G1- but not in G2-phase cells, suggesting that BEZ235 primarily affects non-homologous end joining. This finding was confirmed using a DSB repair reporter gene assay and could be attributed to an impaired phosphorylation of DNA-PKcs (S2056). Cellular radiosensitivity increased strongly after BEZ235 addition in all HNSCC cell lines used, especially when irradiated in the G0 or G1 phase. Our data indicate that targeting the PI3K/Akt/mTOR pathway by BEZ235 with concurrent radiotherapy may be considered an effective strategy for the treatment of HNSCC, regardless of the HPV and Akt status.

## 1. Introduction

Head and neck cancer affect more than 830,000 individuals worldwide, with a cure rate of about 50% [1]. Since the 1990s, locally advanced head and neck squamous cell carcinoma (HNSCC) has been treated by concomitant cisplatin-based radiochemotherapy in the (neo)adjuvant setting, with or without radical surgery [2]. The identical treatment is applied to both HNSCC entities, which are subdivided according to their etiology. Human papillomavirus (HPV)-positive (HPV pos.) HNSCC develops following infection with human papillomaviruses (HPVs) of high-risk genotypes, while HPV-negative (HPV neg.) HNSCC is mainly driven by tobacco and alcohol abuse. HPV infection is a positive prognostic marker indicating an overall survival of about 80% for patients with HPV pos. tumors after a 8-year follow-up, compared to about 30% for HPV neg. patients [3]. Treatment escalation would be appreciated to improve the outcome of HPV neg. HNSCC, but side effects are already severe. Therefore, innovative molecular targeting strategies are needed with the aim to reduce the side effects for both entities and to also increase the outcome, especially of patients with HPV neg. HNSCC. 

In this respect, the receptor of the epidermal growth factor (EGFR) was considered to be an ideal target, since its expression was found to be enhanced in more than 80% of HNSCC, and the upregulated protein can be targeted clinically using the inhibitory antibody cetuximab [4]. However, for cetuximab applied in a curative setting in combination with chemoradiotherapy, there is no benefit for patients; recent clinical studies show no improved outcome [2]. One biological explanation might be that EGFR inhibition results in radiosensitization via cell cycle arrests, which is abrogated by fractioned irradiation [5,6]. 

The phosphoinositide 3-kinase/protein kinase B/ mammalian target of rapamycin (PI3K/Akt/mTOR) pathway shows a high frequency of mutation in HNSCC with up to 62% of cases carrying activating genetic changes in PI3K in both HNSCC entities [7,8,9]. While being positively stimulated by receptor tyrosine kinases (RTKs), the phosphatase and tensin homolog (PTEN) suppresses signaling via the pathway. Since RTKS and PTEN are also frequently deregulated, the PI3K pathway is hyperstimulated in HNSCC [10]. PI3K/Akt/mTOR signaling regulates cell growth and proliferation, apoptosis, the DNA damage response [11], and acts as a main driver of cellular survival mechanisms after irradiation [12]. Radioresistance is achieved via Akt1, which affects DNA double-strand break (DSB) repair after being activated by phosphorylation. Depletion of Akt was reported to impair both homologous recombination (HR) [13] as well as non-homologous end joining (NHEJ) [14], the latter in dependence of DNA-dependent protein kinase, catalytic subunit (DNA-PKcs) [12]. 

The small molecule NVP-BEZ235 (BEZ235) is considered to be a very potent inhibitor, specifically suppressing the activation of PI3K and mTOR, which both carry a PI3K catalytic domain with high homology. The protein stability of downstream targets, such as phosphoinositide-dependent kinase-1 (PDK1) and Akt, remains unaffected, while Akt phosphorylation at S473, a target of the mTOR complex 2, is decreased [15]. BEZ235 is a pan-class I PI3K inhibitor, diminishing kinase activity by occupying the ATP-binding cleft in the catalytic domain of these enzymes, and this mechanism can also affect ATM, ATR, and DNA-PKcs due to their homologous protein conformation [16]. Several publications confirm not only the strong anti-proliferative effect of the drug on PI3K pathway-mutated solid tumors, but also show its radiosensitizing potential. However, the efficacy of BEZ235 towards the affected enzymes remains a matter of debate, and seems to depend on drug concentration, timing, tumor entity, and genomic background [17,18,19,20,21,22,23]. 

In the current study, we examined whether BEZ235 can be used to increase radiosensitivity in both HNSCC etiologies. The study was performed with five HPV neg. and five HPV pos. HNSCC cell lines, with the second group showing an enhanced cellular radiosensitivity [24], which is attributed to an impaired DSB repair [25,26]. BEZ235 strongly increases the cellular radiosensitivity of all HNSCC cell lines, independently from HPV status. This effect is cell cycle dependent, with much stronger radiosensitization observed in G0- or G1-phase cells, when compared to S-phase cells. In line with the data, radiosensitization primarily resulted from a suppressed repair of radiation-induced DSBs by NHEJ, as demonstrated by an excellent correlation between the increase in radiosensitivity and the decrease in DSB repair, as measured for G1 cells.

## 2. Results

### 2.1. Akt Status in HPV Negative and Positive HNSCC Cells Shows Similar Variation 

**Figure 1:**
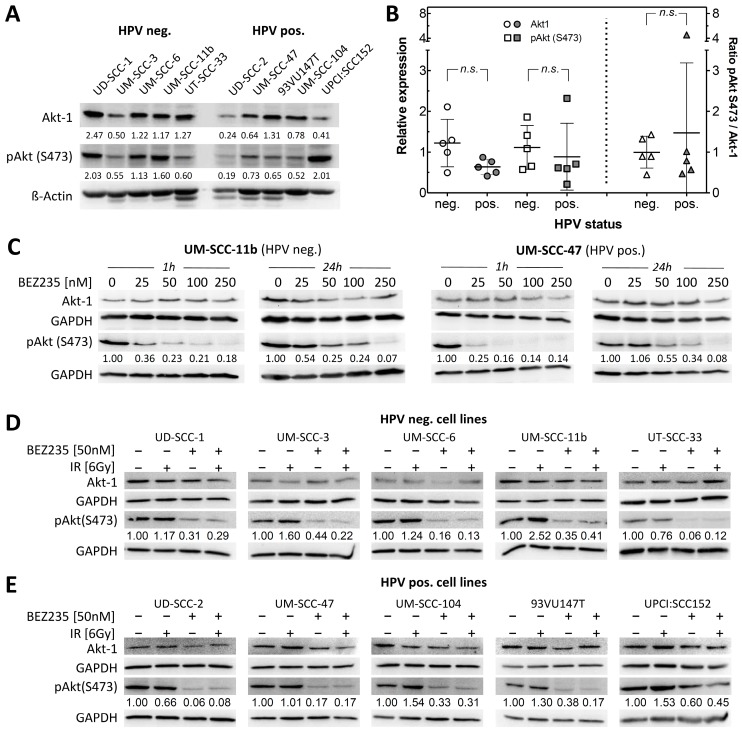
Akt status and down-regulation of p-Akt by BEZ235 in human papillomavirus (HPV)-negative (neg.) and positive (pos.) Head and neck squamous cell carcinoma (HNSCC) cell lines. (**A**) Akt and pAkt expression as determined by Western blot. (**B**) Variance of the Akt and pAkt (S473) expression as well as the respective ratio of pAkt/Akt. (**C**) Downregulation of pAkt by BEZ235 in UM-SCC-11b and UM-SCC-47 cells. Cells were incubated with BEZ235 for 1-h or 24 h at different concentrations before lysed for Western blot. Amount of pAkt was expressed relative to the control level. (**D**,**E**) Downregulation of radiation-induced activation of pAkt by BEZ235 in HPV neg. (**D**) and pos. (**E**) cell lines. Cells were incubated with BEZ235 (50 nM) for 2 h before irradiation with 6 Gy. For Western blot analysis, cell extracts were obtained 1-h after irradiation. Amount of pAkt as expressed relative to the control level.

The serine/threonine protein kinase Akt harbors two phosphorylation sites, which determine the activation level of the protein. Residue Thr308 is a target of PDK1 [27], which becomes activated by PI3K. Residue Ser473 is activated by mTORC2 [28,29,30] and DNA-PK [14]. For the ten HNSCC cell lines studied, protein expression levels of Akt as well as pAkt were analyzed by Western Blot (Figure 1A). Both Akt as well as pAkt varied between the cell lines by a factor of up to five, but with no significant difference between the two entities (Figure 1B, left). Determination of the pAkt/Akt ratio showed almost identical values for both entities, indicating comparable activities in all cell lines examined (Figure 1B, right). 

### 2.2. BEZ235 Suppresses Akt Phosphorylation on Ser473 Independent of HPV Status 

Since BEZ235 inhibits both PI3K (which activates PDK1) and mTOR (which is part of the mTORC2 complex), both Akt phosphorylation sites are affected by the drug. As for HNSCC, Ser473 was found to be the relevant site, having implications in treatment outcome and prognostic value in HNSCC patients [31]; we focused on that site specifically. Single doses of BEZ235 applied for 1-h inhibited the phosphorylation at Ser473 3-4-fold, at concentrations as low as 25 nM, persistence over 24 h (Figure 1C) was reached for concentrations of 50 nM and higher, as seen for both an HPV pos. (UM-SCC-47) as well as an HPV neg. (UM-SCC-11b) cell line. For further experiments, an incubation with 50 nM BEZ235 for 2 h prior further treatment was used. 

Radiation is known to increase Akt phosphorylation in various tumor entities, which may cause therapy resistance [32]. To analyze Akt phosphorylation upon irradiation, cells were treated with 6 Gy and harvested for Western Blot 1-h thereafter. Upon irradiation a very heterogeneous pattern was seen for pAkt with an increase in only a few cell lines (Figure 1D,E). BEZ235 treatment, which was applied 2 h before irradiation, could not only reduce basal phosphorylation activity similar to that shown in Figure 1C, but also abolish radiation-induced activation of Akt. These results suggest that BEZ235 inhibits endogenous and exogenous PI3K/Akt pathway activation in HNSCC, independent of HPV status. 

### 2.3. BEZ235 Leads to a Moderate G1 Arrest without Affecting Radiation-Induced Apoptosis

**Figure 2:**
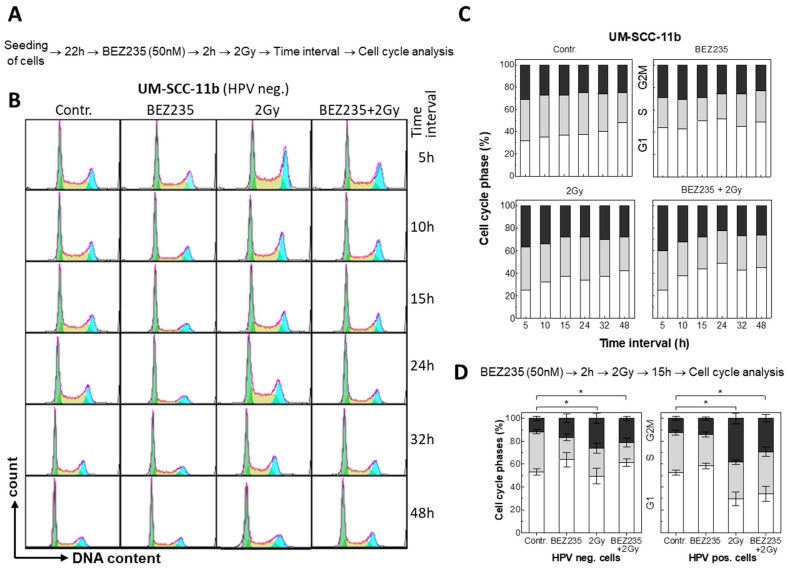
Effect of BEZ235 on cell cycle distribution in HPV neg. and pos. HNSCC cell lines; 22 h after seeding, cells were incubated with BEZ235 for 2 h prior to 2 Gy irradiation, followed by an incubation from 5 h to 48 h. Quantification of cell cycle distribution was performed by flow cytometry of propidium-iodide-stained cells. (**A**) Treatment schedule. (**B**) Cell cycle distribution of UM-SCC-11b cells as determined 5 h to 48 h after treatment. (**C**) Percentage of cell cycle phases of UM-SCC-11b cells taken from (**B**). (**D**) Summary of the cell cycle distribution of all five HPV neg. and pos. HNSCC cell lines as determined 15 h after treatment. Significant differences in G1 distribution between conditions are shown with asterisks (*p*-value < 0.05).

The PI3K/Akt/mTor signaling pathway is required for G1/S progression [33] and inhibition of the pathway will induce G1 arrest. In agreement with that, inhibition with BEZ235 was found to result in a moderate increase in G1-phase cells and a moderate decrease in S-phase, as shown for HPV neg. UM-SCC-11b cells (Figure 2A–C). The G2/M-phase population was not affected. When combined with irradiation, this effect was found to overlay with the radiation-induced G2 arrest. This is also seen for all ten HNSCC cell lines when cell cycle distribution was measured 15 h after irradiation (Figure 2D, Figure S1).

Treatment of HPV pos. cell line UM-SCC-104 by BEZ235 alone was found to cause an increase in the number of apoptotic cells by only one percent as detected by Annexin V-FITC staining (Figure S2). When combined with irradiation, only an additive effect was seen. Similar data were obtained for the other cell lines (data not shown) indicating that BEZ235 does not alter the radiation-induced apoptosis. 

### 2.4. BEZ235 Strongly Inhibits the Repair of Radiation-Induced DSBs Only in G1 

**Figure 3:**
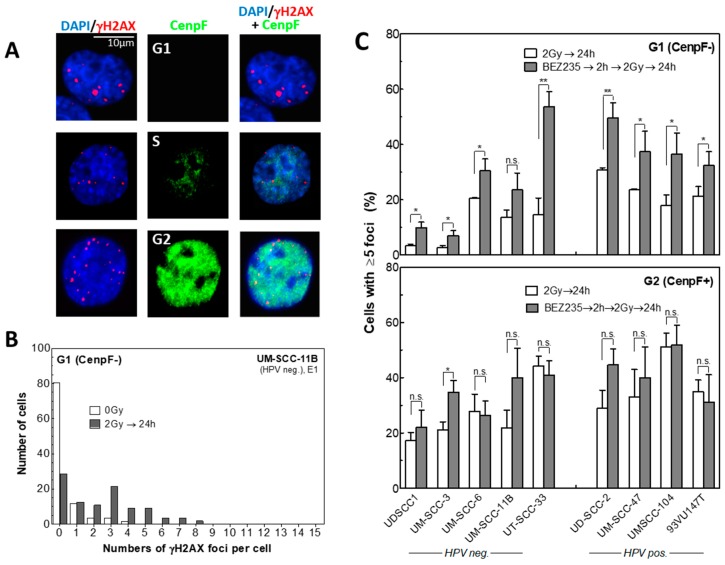
Effect of BEZ235 on double-strand break (DSB) repair in HPV neg. and pos. cell lines. Exponentially growing cells were incubated with 50 nM BEZ235 for 2 h prior to 2 Gy irradiation and examined after 24 h. (**A**) γH2AX foci detection and co-staining of centromere protein F (CenpF) to discriminate between G1 (CenpF-, negative), and G2 (CenpF+, positive) phase cells. Scale bar is shown at the image. (**B**) Distribution of the number of residual γH2AX foci per nucleus, as determined for UM-SCC-11b cells 24 h after 0 Gy or 2 Gy irradiation in G1 cells. (**C**) Percentage of cells with ≥5 γH2AX foci per nucleus in G1 (upper panel) cells or in G2 (lower panel) cells. Significant differences between conditions are shown with one (p-value < 0.05) or two asterisks (p-value < 0.01).

Akt is a known key player in DNA repair and reported to stimulate both HR and NHEJ [12,13]. To analyze DNA DSB repair upon BEZ235 treatment, we investigated γH2AX foci 24 h after irradiation. To distinguish between the G1 and G2 phase, cells were additionally stained for the centromere protein F (CenpF) (Figure 3A) as described previously [34]. Unfortunately, this analysis could not be performed for UPCI: SCC152 cells because of an extremely high level of unspecific γH2AX foci. For unirradiated cells, generally less than 5 foci are seen per cell, with most cells showing no or only one focus (Figure 3B). Therefore, data of irradiated cells were quantified by using the percentage of cells with ≥5 γH2AX foci per nucleus. 

After treatment with 2 Gy, the fraction of cells with ≥5 residual γH2AX foci measured per cell was generally higher for HPV pos. cells. This is well in line with previous data showing that HPV pos. HNSCC cells are defective in DSB repair [24,25]. When this fraction of residual foci was plotted vs. the Akt status, no association was seen for Akt, and only trends for pAkt, or the ratio of both (Figure S3A).

Pretreatment with BEZ235 2 h prior to irradiation was found to cause an increase in the amount of residual γH2AX foci, as observed for all cell lines, except for UM-SCC-11b cells. However, this increase was only seen for cells analyzed in the G1-phase (Figure 3C, upper panel). On the contrary, no clear effect was observed for G2-phase cells (Figure 3C lower panel, CenpF+) where, except for UM-SCC-3, no significant changes in residual repair foci were detected. Moreover, no association was seen between the increase in residual foci and the Akt status, when plotted, vs. the different parameters (Figure S4A).

Together these data indicate that BEZ235 inhibits DSB repair in both HPV neg. and pos. cell lines by blocking a repair process, which is predominantly active in G1-phase cells. 

### 2.5. BEZ235 Suppresses NHEJ Due to an Impaired Phosphorylation of DNA-PKcs at S2056 

**Figure 4:**
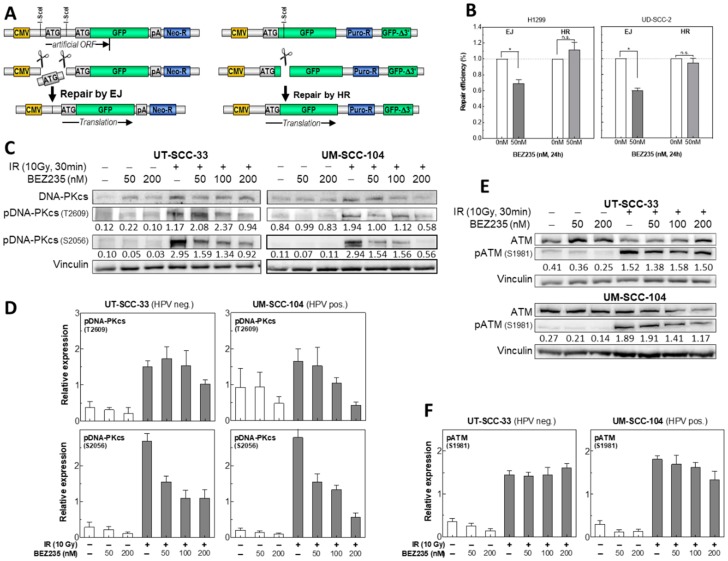
Effect of BEZ235 on DSB repair pathways. (**A**) Double-Strand Break (DSB) repair constructs for end-joining (EJ) and for homologous recombination (HR). (**B**) Effect of BEZ235 on non-homologous end joining (NHEJ) and HR. H1299 and UD-SCC-2 cells with stably integrated repair plasmids were incubated with BEZ235 (50 nM) for 24 h before repair activity was determined via flow cytometry. Values are normalized to the activity measured for untreated cells. Significant differences between conditions are shown with asterisks (p-value < 0.05). (**C**,**D**) Effect of BEZ235 on the radiation-induced activation of DNA-PKcs. Cells were incubated with BEZ235 (0–200 nM) for 2 h before irradiated with 10 Gy. Protein extracts of UT-SCC-33 and UM-SCC-104 cells were prepared 30 min after irradiation and level of DNA-PKcs, and pDNA-PKcs at T2609, as well as S2056 were measured using Western blotting. (**E**,**F**) Effect of BEZ235 on the radiation-induced activation of ATM. Cells were incubated with BEZ235 (0–200 nM) for 2 h before irradiation with 10 Gy. Protein extracts of UT-SCC-33 and UM-SCC-104 cells were prepared 30 min after irradiation and the level of ATM and pATM (S1981) was measured using Western blotting.

To analyze the DSB repair pathways after BEZ235 treatment in more detail, specifically designed repair plasmids were integrated into the genome of H1299 and UD-SCC-2 cells [35,36]. For NHEJ, an artificial open reading frame (ORF) is created carrying two start codons with one being flanked by I-SceI enzyme recognition sites. Cleavage of one transcriptional start site ATG, and rejoining of ends leads to expression of green fluorescent protein (GFP) (Figure 4A). For HR, full-length GFP is inactivated by insertion of the I-SceI recognition site. Upon cleavage and DSB formation, repair by HR utilizing the sequence of the 3′-truncated copy (ΔGFP) installs a functional GFP (Figure 4A). As determined by flow cytometric measurement, treatment with 50 nM BEZ235 for 24 h was found to reduce NHEJ by 30–40% with virtually no effect, or even a small enhancement for HR (Figure 4B). Such a slight increase in HR was also seen when recording the effect of BEZ235 on the Rad51 formation in irradiated UM-SCC-11b cells (Figure S5).

In NHEJ-mediated repair of DSBs, DNA-PKcs is a crucial player, and Akt was described to be involved in activating DNA-PKcs after irradiation-induced DNA damage [12,37]. Phosphorylation of the residues T2609 and S2056 is activating DNA-PKcs, and an indicator for efficient DSB repair capacity. At both sites, phosphorylation increases upon irradiation with 10 Gy as determined for HPV neg. (UT-SCC-33), as well as HPV pos. cells (UM-SCC-104; Figure 4C,D). The dose of 10 Gy was used in order to achieve a significant increase, especially for phosphorylation of DNA PKcs at T2609. For neither cell line treatment with 50 nM, BEZ235 was found to impair the radiation-induced phosphorylation at T2609 (Figure 4C,D). A significant reduction was only seen at a higher concentration of 200 nM BEZ235. In contrast, at S2056, a strongly diminished phosphorylation was already obtained at a concentration as low as 50 nM. 

For control, we also examined the effect of BEZ235 on the phosphorylation of ATM at Ser1981. For UT-SCC-33 no, and for UM-SCC-104 cells, only a modest reduction was seen when treated by 200 nM BEZ235 (Figure 4E,F). Since ATM is involved in HR [38], this finding is in line with the observation made above that BEZ235 was found to have no effect on HR. Overall, these data indicate that administration of 50 nM BEZ235 inhibits DSB repair by suppressing NHEJ because of a retarded activation of DNA-PKcs at S2056.

### 2.6. BEZ235 Causes Radiosensitization, Which Is Most Pronounced in G0 and G1 

**Figure 5:**
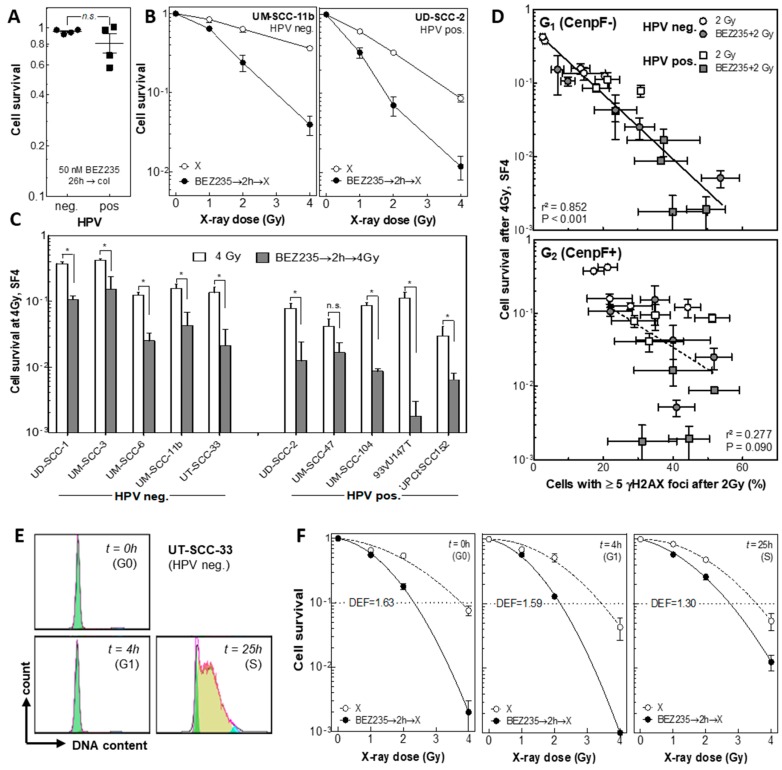
Effect of BEZ235 on cellular radiosensitivity of HPV neg. and pos. cell lines. 22 h after seeding cells were incubated with BEZ235 (50 nM) for 2 h, then irradiated. After 24 h BEZ235 was removed and colonies were allowed to grow up to 21 days. (**A**) Effect of BEZ235 on cell survival. (**B**) Effect of BEZ235 on UM-SCC-11 and UD-SCC-2 cells irradiated with X-ray doses up to 4 Gy. (**C**) Effect of BEZ235 on cell survival in four HPV neg. and pos. cell lines irradiated with 4 Gy (SF4). Significant differences between conditions are shown with asterisks (p-value < 0.05). (**D**) Association between cell survival and DSB repair after irradiation alone or in combination with BEZ235. Percentage of cells with ≥5 foci as determined either for G1 or G2 phase cells (Figure 3C,D) was plotted vs. the cell survival measured at 4 Gy (SF4). Data were analyzed by linear regression. (**E**,**F**) Effect of BEZ235 on cellular radiosensitivity in dependence of cell cycle. (**E**) Cell cycle distribution of UT-SCC-33 cells 0, 4, and 25 h after re-stimulation of quiescent cultures representing G0, G1, and S-phase population. (**F**) Effect of BEZ235 on radiosensitivity of UT-SCC-33 cells irradiated either in G0, G1, or S-phase.

The effect of BEZ235 on cellular radiosensitivity was determined via colony formation assay. To avoid biases caused by changes in proliferative capacity after BEZ235 treatment, colonies were stained at different time intervals after treatment, ranging from 12 up to 28 days, to achieve colonies of similar size for all treatment conditions (Figure S5). Single treatment with 50 nM BEZ235 for 26 h showed only marginal effects on clonogenicity with no significant difference between HPV neg. and pos. HNSCC cells (Figure 5A). For asynchronously growing UM-SCC-11b (HPV neg.) as well as UD-SCC-2 (HPV pos.) cells, a strong radiosensitization was obtained when cells were pretreated with 50 nM BEZ235 (Figure 5B). This strong and significant reduction of cellular survival was confirmed in all HPV pos. and HPV neg. cell lines used in this experiment, indicating an independence of the radiosensitizing effect of BEZ235 from HPV status (Figure 5C). Cellular radiosensitivity after irradiation alone, or in combination with BEZ235, was also not associated with the Akt status (Figures S3B and S4B). 

Correlation of cell survival at 4 Gy with the percentage of cells with ≥5 γH2AX foci reveals a significant relationship for G1- but not for G2-phase cells (Figure 5D, r^2^ = 0.840, *p* < 0.001 vs. r^2^ = 0.187, *p* = 0.095). This finding demonstrates that the radiosensitization achieved by BEZ235 is due to the reduced DSB repair occurring in G1-phase cells. 

To verify this data, radiosensitization was also studied in dependence of cell cycle. UT-SCC-33 cells were synchronized in G0/G1 phase by confluent growth and then reseeded to obtain a G0-, G1-, and S-phase population (Figure 5E). The radiosensitization mediated by BEZ235 was stronger for G0- and G1-phase cells than for S-phase cultures, with dose enhancement factors (DEF), as calculated at 10% survival of 1.63, 1.59, and 1.39, respectively (Figure 5E). Overall these data indicate that the radiosensitization achieved by BEZ235 can be attributed to its inhibitory effect on DSB repair via a depressed NHEJ.

## 3. Discussion

Therapeutic failure in the treatment of HNSCC is often attributed to an inherent radioresistance of the tumor cells. Intrinsic factors, such as deregulation of the PI3K/Akt/mTOR pathway, as well as extrinsic factors, such as irradiation-induced upregulation of Akt signaling, play major roles in resistance towards therapy. The effect of mono-treatment with the dual inhibitor BEZ235 towards this pathway was already investigated in several studies, including phase I clinical trials, but with no substantial response [39,40]. More benefit might be expected when BEZ235 is combined with radiotherapy, since several published preclinical studies confirm in vitro, as well as in vivo, an increase in radiosensitivity for various tumor entities, such as glioblastoma [19,20,21], colorectal [18,41], lung [17] and breast cancer [42], as well as HNSCC [21,23]. 

The experiments presented here were performed with ten HNSCC cell lines, which were previously shown to be a good preclinical model to reflect the clinical response of these tumors, with HPV pos. HNSCC, exhibiting a much better response towards combined radiochemotherapy [24,25,26,43,44]. BEZ235 was found to abrogate basal phosphorylation of Akt1 at S473, at concentrations as low as 50 nM, and also to inhibit the radiation-induced activation of Akt1 at this site. Similar results were obtained by others [19,45]. BEZ235 did not substantially increase the number of apoptotic cells, with only an additive effect when combined with radiation, as also observed in other reports [22,46]. However, in one publication, an increase in apoptosis was also seen, which may depend on the mutational status of specific genes, such as Kras [17,42]. BEZ235 induced a moderate G1-arrest in all HNSCC cell lines with slightly stronger levels for HPV neg. cells. When combined with radiation, an overlay of the BEZ235-induced G1-arrest and the radiation-induced G2-arrest was found. 

BEZ235 was measured to have a pronounced effect on the repair of radiation-induced DNA DSBs as recorded via the γH2AX foci assay. Treatment with 50 nM BEZ235 prior to an exposure with 2 Gy resulted in a significant increase in the percentage of cells with ≥5 residual foci, as measured 24 h after irradiation. It is already known that BEZ235 may impair repair of radiation-induced DSBs [19,20,21,46]. However, it is now shown here for the first time that this effect is cell cycle dependent, with BEZ235 primarily affecting DSB repair in G1- but not G2-phase cells. In line with these data, we found that BEZ represses NHEJ, which is the major DSB repair pathway acting in G1. In contrast, no effect or even a slight increase was seen for HR, which is only active in late S and G2 [47]. In line with this, a slight increase in Rad51 formation was seen for irradiated UM-SCC-11b cells when pretreated by BEZ235. Such a shift of DSB repair to HR was also reported for other cells when NHEJ was depressed [36].

NHEJ is performed by a multi-enzymatic complex with DNA-PKcs as a key enzyme. Upon radiation, DNA-PKcs is phosphorylated at several serine and threonine sites organized in defined clusters, with the two most important sites at S2056 and T2609 [48]. Radiation-induced activation of S2056 is caused by autophosphorylation of DNA-PKcs, while phosphorylation at T2609 is mediated by activated ATM [49]. It was found that pretreatment by 50 nM, BEZ235 strongly inhibits the radiation-induced phosphorylation of DNA-PKcs at S2056. In contrast, for the phosphorylation of DNA-PKcs at T2609, as well as of ATM at S1981, a significant reduction was only achieved at concentrations as large as 200 nM BEZ235. Therefore, the pronounced reduction in DSB repair and NHEJ, already observed at 50 nM, BEZ235 is considered to result from an impaired autophosphorylation of DNA-PKcs at S2056. A depressed NHEJ, together with a reduced phosphorylation of DNA PKcs at S2056, when X-irradiation was combined with BEZ235, was likewise seen by Mukherjee et al. [19]. However, this group also observed that BEZ235 inhibits HR in 1BR3 fibroblasts by studying Rad51 foci formation, or in MCF7 cells, by using a respective DSB repair construct. These results are in clear contrast to the data shown here for HNSCC cells, as outlined above, indicating that the effect of BEZ235 might depend on the cell line used. 

For all ten HNSCC cell lines used, pre-treatment by 50 nM BEZ235 was shown to result in a strong radiosensitization. Such an effect was also observed for other tumor cell lines and especially for glioblastoma cells [18,20,21,42,46,50]. It is now shown here that this sensitization is equally effective for HNSCC cell lines independent of their HPV or Akt status. In agreement with the previous reports, our data suggest that this radiosensitization results from a depressed DSB repair, and it is now demonstrated that this is primarily due to a depressed DSB repair performed in the G1-phase by NHEJ. This was demonstrated by the excellent correlation between cell survival at 4 Gy, and the respective increase in the percentage of cells with ≥5 residual foci. In line with this, radiosensitization by BEZ235 was found to be most prominent in G0 and G1 cells, where DSB repair is known to be performed primarily by NHEJ [47]. This finding is also in agreement with our recent observation that HPV pos. HNSCC cells are deficient in HR [26]. In case BEZ235 also suppresses HR, clearly less radiosensitization by BEZ235 should be expected for HPV pos. HNSCC cells when compared to HPV neg. HNSCC cell lines, which, however, was not observed. 

Overall, our data indicate that BEZ235 can be considered an excellent tool to achieve a pronounced radiosensitization in both HPV pos. and neg. HNSCC. So far, these tumors are mostly treated by radiochemotherapy using cisplatin (CPPD) or similar compounds. This combination, however, is associated with strong side effects also limiting a further enhancement by this treatment. For HPV pos. HNSCC, great effort is made to find a strategy for de-escalation, while for HPV neg. HNSCC, there is still a great need to improve tumor control. The in vitro data available so far indicate that a benefit might be expected when BEZ235 is combined with radiotherapy. As shown here, the drug is already effective at low concentration when given two hours prior to irradiation, and should be applied, especially to slowly proliferating HNSCC tumors, with most tumor cells being either in G0 or G1. This concept has to be validated in further studies. 

## 4. Materials and Methods 

### 4.1. Cell Lines and Culture Conditions 

Experiments were performed with five HPV neg. (UD-SCC-1, UM-SCC-3, UM-SCC-6, UM-SCC-11b, UT-SCC-33) and five HPV pos. (UD-SCC-2, UM-SCC-47, UM-SCC-104, 93VU147T, UPCI: SCC152) HNSCC cell lines and H1299 cells (ATCC, Wesel, Germany). Cell lines were kindly provided by T. E. Carey (UM-SCC-6, UM-SCC-11b, UM-SCC-104, UM-SCC-47), University of Michigan, United States; H. Joenje (93-VU-147T), VU Medical Center, Amsterdam, The Netherlands; R. A. Grenman (UT-SCC-33), Turku University, Finland; H. Bier (UD-SCC-1, UD-SCC-2), University of Munich, Germany; Susanne M. Gollin (UPCI: SCC152), University of Pittsburgh, United States. The authentication of cell lines was verified using Short Tandem Repeats (STR) analysis at The German Resource Centre for Biological Material (DSMZ) [51]. All cells were cultured in RPMI-1640 medium (Sigma Aldrich, Munich, Germany) supplemented with 10% fetal bovine serum (Biochrom, Berlin, Germany), 2 mM L-glutamine, 1% non-essential amino acids. Cultures were maintained at 37 °C in a humidified 5% CO2 atmosphere, and routinely tested for mycoplasma contamination [52].

### 4.2. Radiation and BEZ235 Treatment 

Cells were irradiated with X-rays using a Precision X-RAD 320ix biological irradiator (Precision X-Ray, North Branford, CT, USA) at 320 kV and 10 mA, dose rate of 1.2 Gy/min, Thoräus filter 0.5 mm Cu+ 0.5 mm Al. Absolute dose measurements confirmed the applied doses.

NVP-BEZ235 (Selleckchem; PubMED CID: 11977753) was applied in a final concentration of 50 nM in DMSO 2 h prior to irradiation, and removed 24 h later, if not stated otherwise.

### 4.3. Clonogenic Assay 

Clonogenicity was analyzed by colony formation assay as described previously [24]. Cells were left to grow until the colonies of the treatment arm had reached equal colony size to the control arm (approximately 10–28 days; Figure S6) and were then fixed and stained for colony counting (≥50 cells). The surviving fractions of irradiated samples (SF) were normalized to the plating efficiency of the respective non-irradiated control and clonogenic surviving fractions calculated. Survival curves were fitted to the linear-quadratic model

SF = exp − [αD + βD2],(1)

according to a least square fit (GraphPad Prism 5.0 software). Each experiment was done in biological triplicates with a minimum of three independent repetitions.

### 4.4. Cell Cycle Distribution 

Cells were dissociated with Accutase, fixed with 70% ice-cold ethanol, and incubated for 30 min in phosphate buffered saline (PBS) containing 200 µg/mL RNase A, 0.1% Triton X-100, and 20 µg/mL propidium iodide. Flow cytometric measurements were performed using an LSR II flow cytometer (Becton Dickinson, Frankfurt, Germany) by analyzing 20,000 cells per sample. Data were processed using FlowJo 7.6 software (Tree Star Inc., San Carlos, CA, USA).

### 4.5. Detection of Apoptosis

Apoptotic cells were assessed using the Annexin V-FITC detection kit (Promokine, Heidelberg, Germany). Cells were detached from the surface, stained with Annexin V-FITC and propidium iodide, according to the manufacturer’s instructions. Annexin V/FITC and PI double-positive cells were considered to be apoptotic, and their portion of all analyzed cells was calculated.

### 4.6. Synchronization of Cells 

Proliferating cells were allowed to grow under optimal conditions until reaching confluency. This quiescent culture (G0) was reseeded to obtain G1 cell culture (time point 4 h) and an early S-phase culture (time point 25 h). Cell cycle distribution was verified by flow cytometric analysis.

### 4.7. Immunofluorescent Microscopy 

Immunofluorescence analysis was performed as previously described [34]. Briefly, cells grown and treated on glass coverslips were washed with PBS and fixed with 4% para-formaldehyde for 10 min. Fixed cells were permeabilized with 0.2% Triton X-100 on ice for 5 min. The cells were incubated with primary antibodies: anti-γ-H2AX (Millipore, Darmstadt, Germany), mouse monoclonal anti-RAD51 antibody (1:1000, clone 14b4, Abcam, Cambridge, UK), and anti-CenpF (Lifespan Biosciences, Seattle, WA, USA); and secondary antibodies: anti-mouse AlexaFluor594 or anti-rabbit Alexa Fluor-488 (Invitrogen, Karlsruhe, Germany). Nuclei were counterstained with 4′,6-diamidin-2-phenylindol (DAPI). Cells were viewed with an IX81 microscope using a 60× Neofluor objective and Xcellence Software (Olympus, Hamburg, Germany). 

γH2AX foci were scored visually in CenpF-negative (G1-phase) and CenpF-positive cells (G2-phase). Generally, non-irradiated cells showed less than five γH2AX foci per cell nucleus. Therefore, data of irradiated cells are presented as the percentage of cells with ≥5 γ-H2AX foci. A minimum of 100 cells in three independent experiments was counted and data are shown as mean values ± standard error of the mean (SEM).

### 4.8. Western Blot Analysis

Following treatment, cells were lysed in ice cold radioimmunoprecipitation assay (RIPA) buffer supplemented with protease inhibitor cocktail and PMSF (AppliChem, Darmstadt, Germany). Lysates were boiled in 1× SDS-PAGE sample buffer (25 mM Tris-HCl, pH 6.8; 10% glycerol, 2% SDS, 2.5% β-mercaptoethanol, 0.005% bromophenol blue) and equivalent amounts of protein were electrophoresed on SDS-PAGE gels. PVDF membranes were blotted with antibodies that recognize Akt, (#2938, 1:1000), p-Akt Thr308 (# 2965, 1:2000), p-Akt Ser473 (# 4060, 1:1500), GAPDH (#2118, 1:1000), (all Cell Signaling, Frankfurt, Germany), ATM (# 1549-1), p-ATM S1981 (# 2152-1, Abcam, Cambridge, UK) and subsequently incubated with anti-rabbit/anti-mouse HRP (horseradish peroxidase)-linked IgG antibodies (1:5000, Millipore, Darmstadt, Germany). Proteins were visualized using enhanced chemiluminescence detection system (Amersham, Munich, Germany). Generally, specific expression was normalized to the mean value of the total blot, or, when specified, to the untreated control. Raw data of Western Blots is provided in Figure S7.

### 4.9. DSB Reporter Gene Assay 

Exponentially growing H1299 cells containing stably integrated copies of the previously described GFP-based HR or NHEJ reporter plasmids pGC or pEJ [30,31], were transfected with an I-SceI expression vector using Fugene HD (Promega, Mannheim, Germany). Six hours post-transfection, the medium was exchanged and supplemented with 50 nM BEZ235 or solvent (DMSO), followed by another exchange, plus supplementation, 24 h post-transfection. At 48 h post-transfection, the cells were harvested and assessed for GFP expression by flow cytometry (FACS Canto with FACS Diva software, Becton Dickinson). The gating of GFP-positive cells was set according to the negative control (Fugene HD and empty vector). Rates of DSB repair (% GFP-positive cells) were normalized to the respective transfection efficiency as determined by transfection with a GFP-expression vector. Results of repair under BEZ235 supplementation are presented as normalized to the respective solvent controls.

### 4.10. Statistical Analysis 

Statistical analyses were performed using GraphPad Prism version 5.04 (GraphPad Software Inc., La Jolla, CA, USA) and statistical significance was assumed for *p*-values < 0.05 using a Mann– Whitney test, or Chi^2^ test, in case of cell cycle analysis. In figures, a significant difference is indicated by * and a hyper-significant difference (*p* < 0.01) by **. 

## 5. Conclusions

In summary, our study revealed that the dual PI3K/mTOR inhibitor BEZ235 is a potent radiosensitizer for both HPV neg. and pos. HNSCC cell lines, with a strong effect especially for G0 and G1-phase cells, indicating its great potential for the treatment of slowly growing HNSCC.

## Supplementary Materials

The following are available online at https://www.mdpi.com/2072-6694/12/2/467/s1, Figure S1: Effect of BEZ235 on cell cycle distribution of HPV neg. and HPV pos. cell lines, Figure S2: Impact of BEZ235 on radiation-induced apoptosis in HPV pos. UM-SCC-104 cells, Figure S3: Association between the expression of Akt and pAkt, Figure S4: Association between the expression of Akt and pAkt, Figure S5: Effect of BEZ235 on Rad51 foci formation after exposure to 2 Gy in UM-SCC-11b cells, Figure S6: Colony formation assay of UD-SCC-2 cells, Figure S7: Western Blot Raw data.
