# Supplementary material for: Dual PI3K/mTOR Inhibitor NVP-BEZ235 Enhances Radiosensitivity of Head and Neck Squamous Cell Carcinoma (HNSCC) Cell Lines Due to Suppressed Double-Strand Break (DSB) Repair by Non-Homologous End Joining
Source: Cancers (Basel). 2020 Feb 18;12(2):467. doi: 10.3390/cancers12020467 (PMC7072694; doi:10.3390/cancers12020467)
Supplement: Supplementary file 1 [file cancers-12-00467-s001.pdf]

Supplementary Materilas

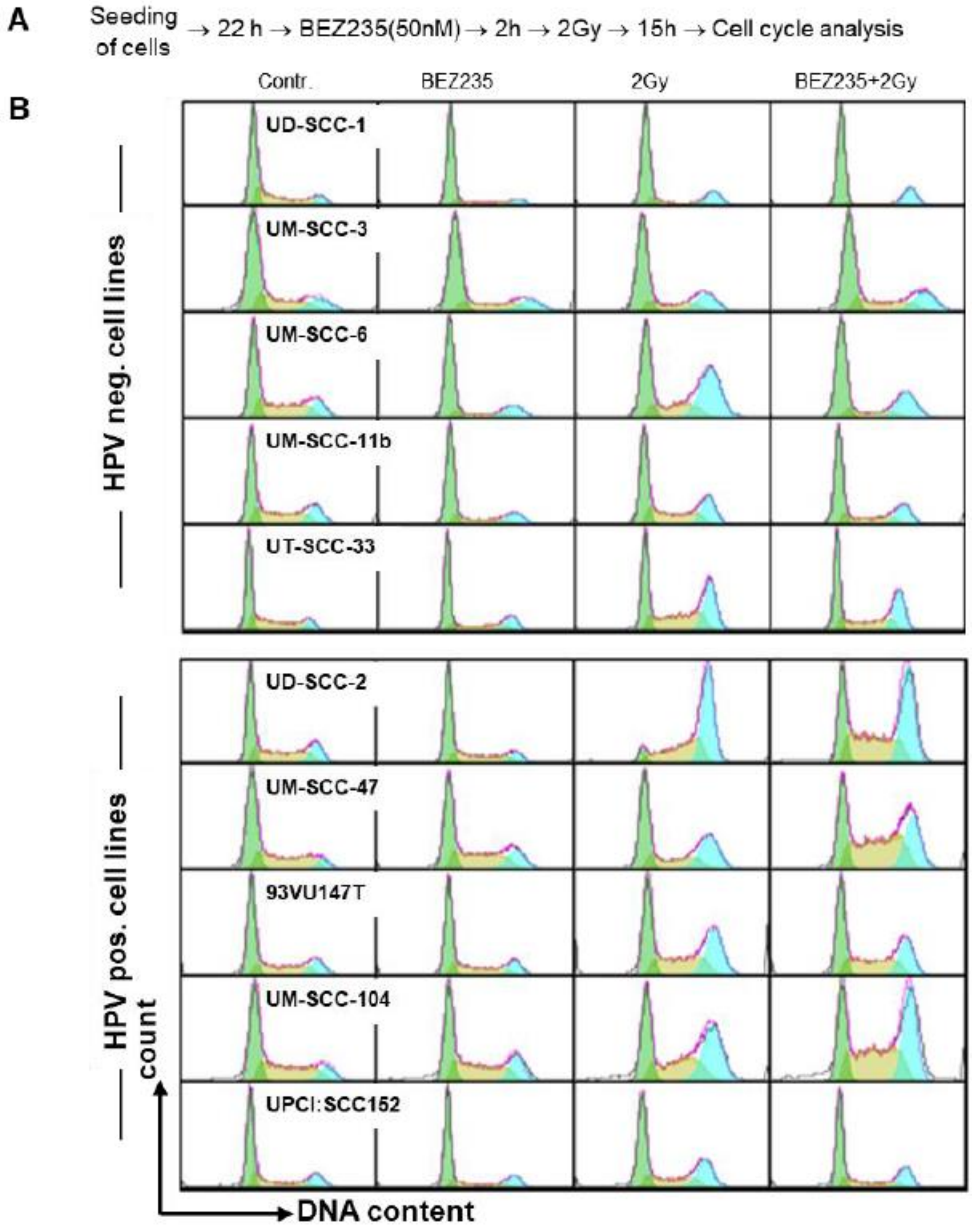

Figure S1. Effect of BEZ235 on cell cycle distribution of HPV neg. and HPV pos. cell lines. 22 h after seeding cells were incubated with BEZ235 (50 nM) for 2 h before irradiated with 2 Gy followed by an incubation for 15 h. **A**) Treatment schedule. **B**) Cell cycle distribution as determined via flow cytometry using PI staining. After treatment with BEZ235 generally a modest G1 arrest is seen. When combined with 4 Gy this G1 arrest overlays with the G2 arrest caused by irradiation.

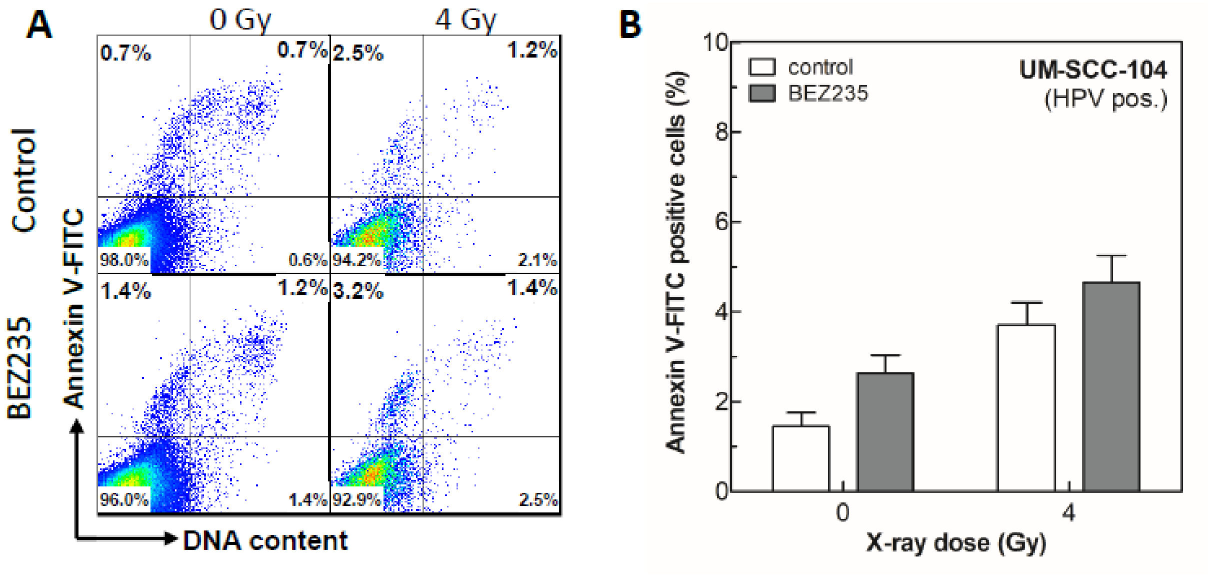

Figure S2. Impact of BEZ235 on radiation-induced apoptosis in HPV pos. UM-SCC-104 cells. **A**) Apoptosis was detected via staining with Annexin V-FITC. **B**) Percentage of Annexin-positive cells as measured 48 h after irradiation with 4 Gy. There was only an additive effect between BEZ235 and 4 Gy.

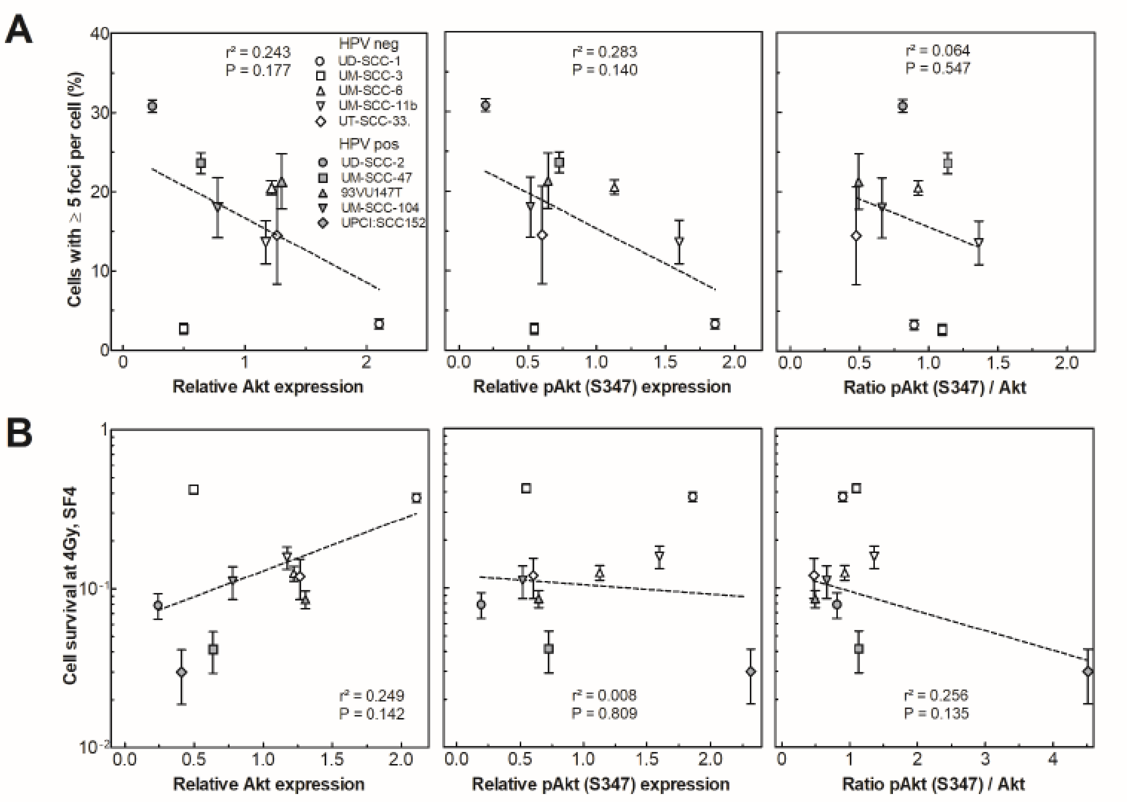

Figure S3. Association between the expression of Akt and pAkt as well as the ratio of these two and **A**) the DSB repair and **B**) the cellular radiosensitivity of HPV neg. and pos. HNSCC cell lines. The DSB repair was expressed as the percentage of cells with ≥5 γH2AX foci per nucleus as measured for G1-phase cells (Figure 3C, upper chart) after irradiation treatment only. The cellular radiosensitivity was expressed as the cellular survival at 4 Gy, SF4 (Figure 5C). Data were analyzed by linear regression. For neither of these parameters a significant association was seen.

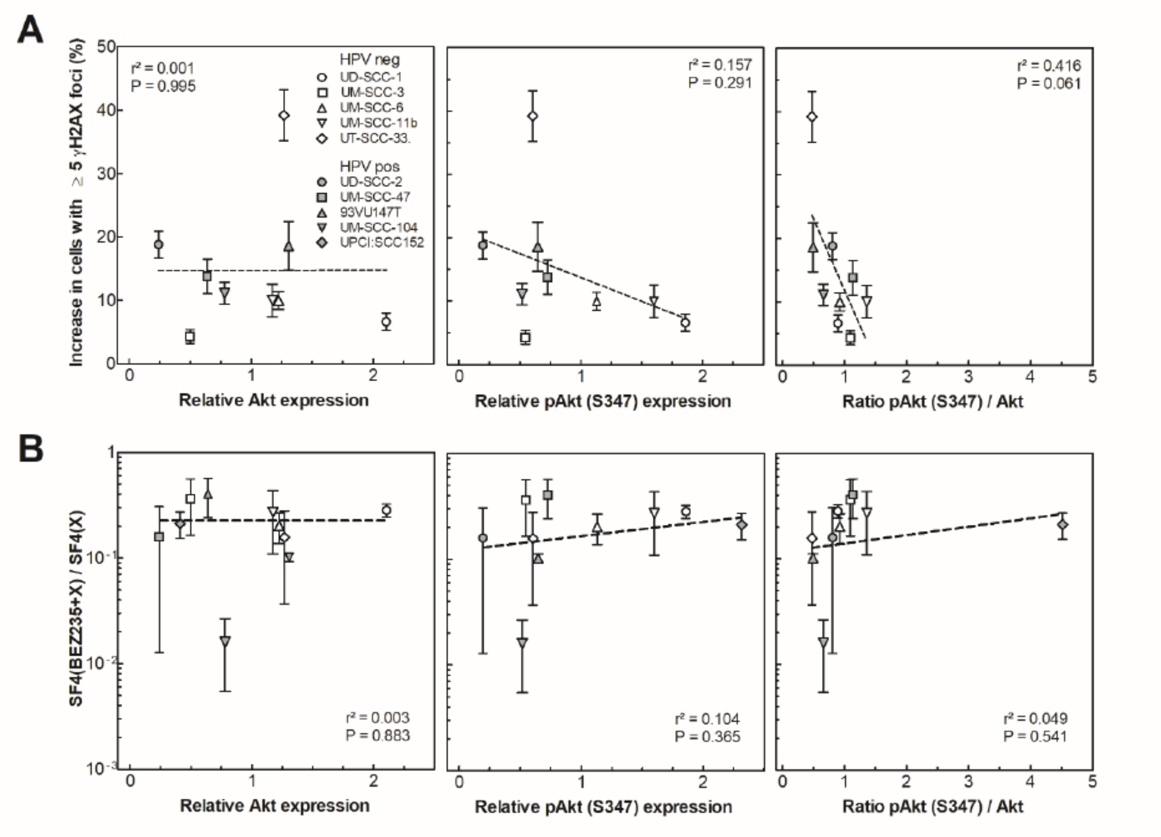

Figure 4. Association between the expression of Akt and pAkt as well as the ratio of these two and the reduction in **A**) DSB or **B**) the radiosensitization caused by 50 nM BEZ235 in HPV neg. and pos. HNSCC cell lines. Reduction in DSB was expressed as the increase in the percentage of cells with ≥5 foci measured for G1-phase cells (taken from Figure 3C) and radiosensitization as the further reduction in cell killing [SF4(BEZ+X)/SF4(X)] achieved when BEZ235 was given prior to 4 Gy (taken from Figure 5C). Data were analyzed by linear regression. For neither of these parameters a significant association with reduction in DSB repair or radiosensitization as caused by BEZ235 was seen.

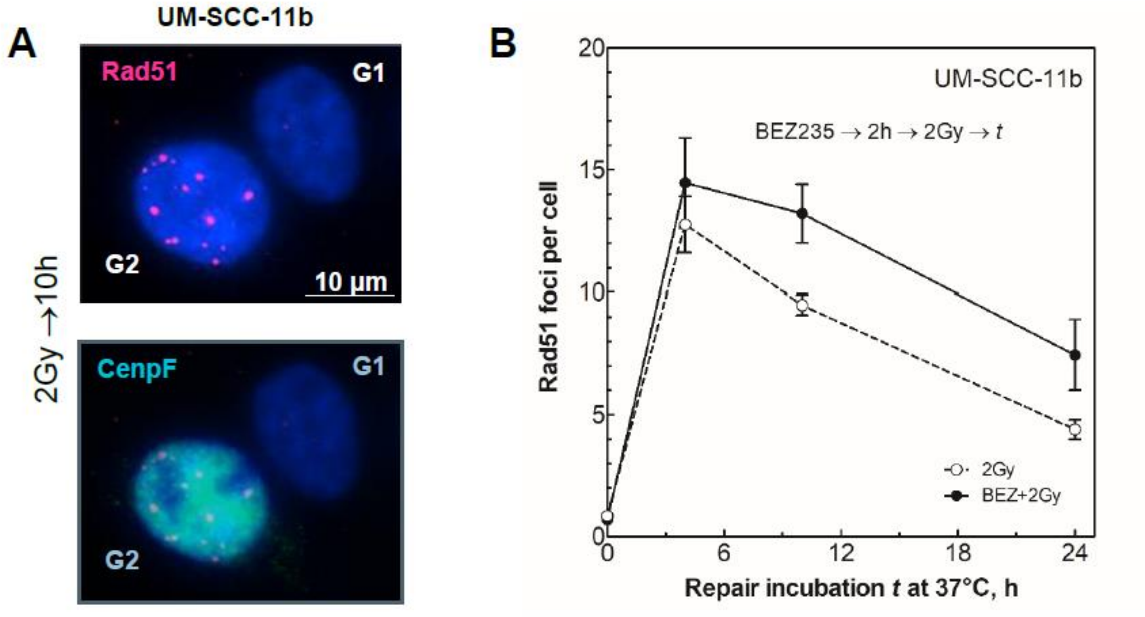

Figure S5. Effect of BEZ235 on Rad51 foci formation after exposure to 2 Gy in UM-SCC-11b cells. Cells were incubated with 50 nM BEZ235 2 h before irradiation, followed by a repair incubation at 37°C up

to 24 h. **A**) Rad51 foci were detected via immunohistochemistry. To distinguish between G1 and G2 phase cells staining with CenpF was used. Scale bar is shown at the image. **B**) Kinetics of Rad51 foci formation. The data demonstrate that Rad51 foci formation was slightly enhanced in UM-SCC-11b cells, when treated by BEZ235 indicating that DSB repair might be shifted to homologous recombination.

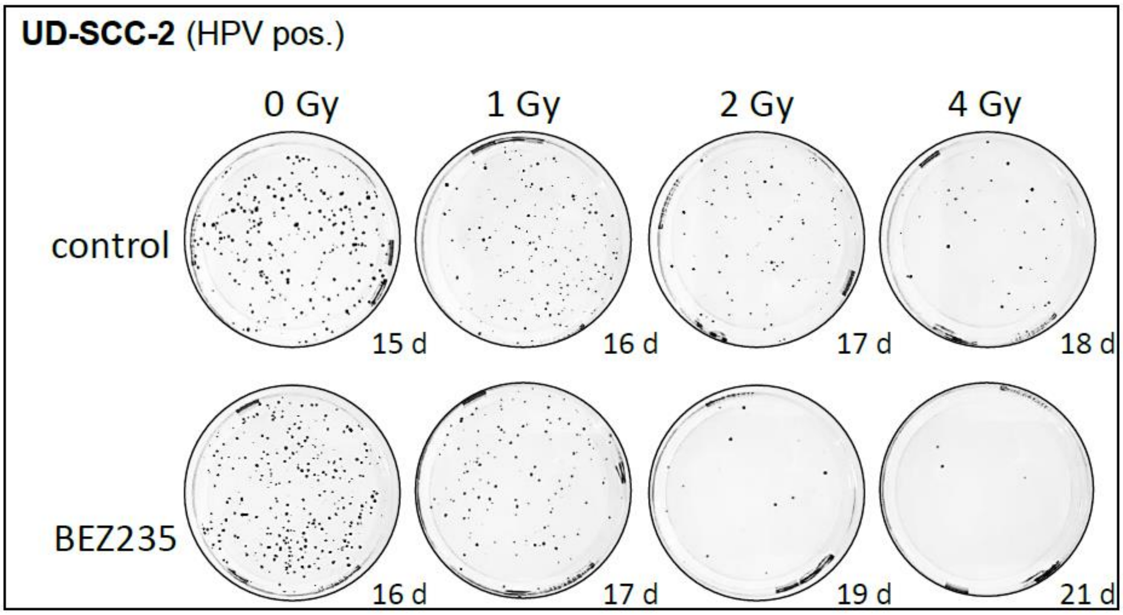

Figure S6. Colony formation assay of UD-SCC-2 cells. 22 h post seeding cells were incubated with BEZ235 (50 nM) for 2 h before irradiated with X-ray doses up to 4 Gy. After irradiation cells were incubated for 24 h before medium was replaced by BEZ235-free medium followed by colony growth varying from 15 up to 21 days to adapt for similar colony sizes.

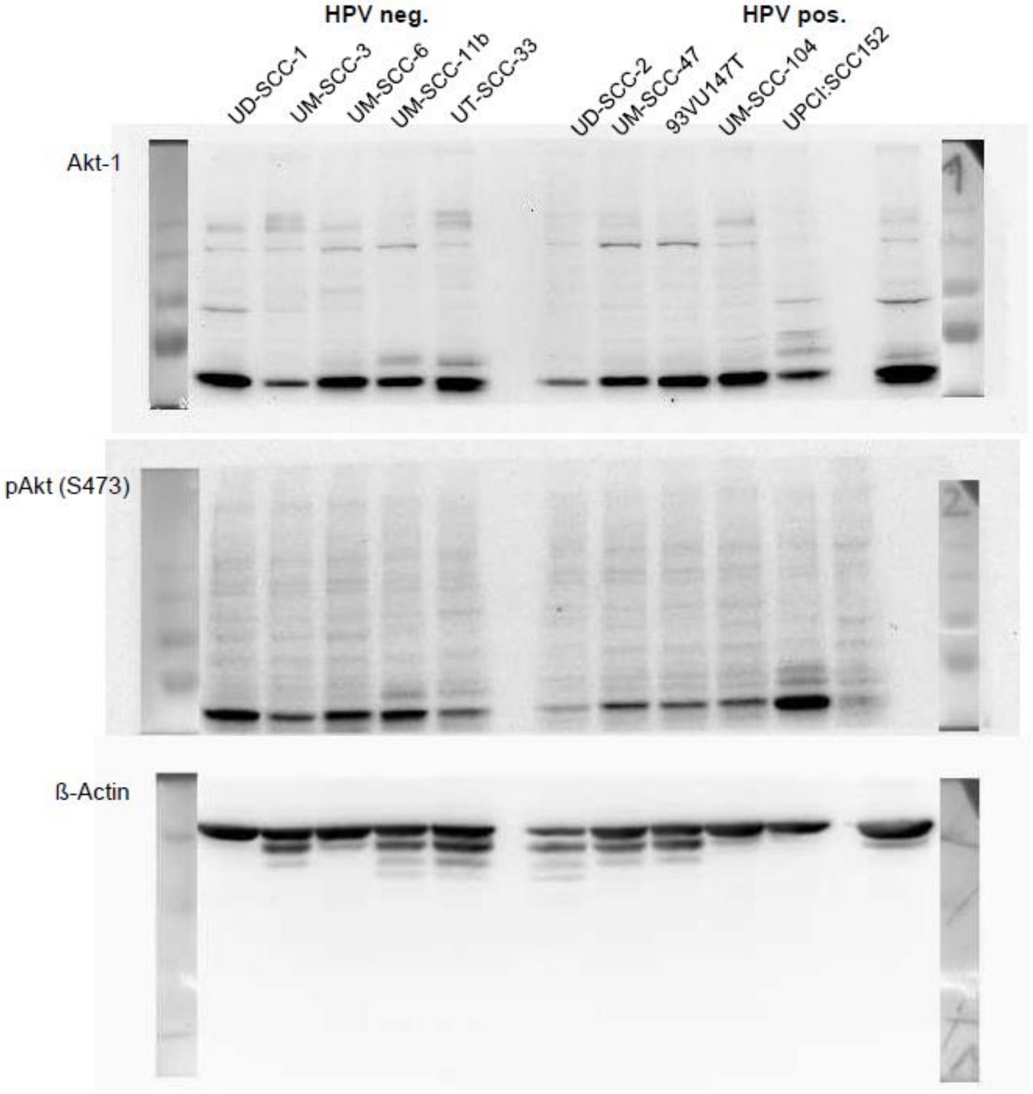

Figure S7. Supplementary data Western Blot raw data corresponding to Figure 1A.

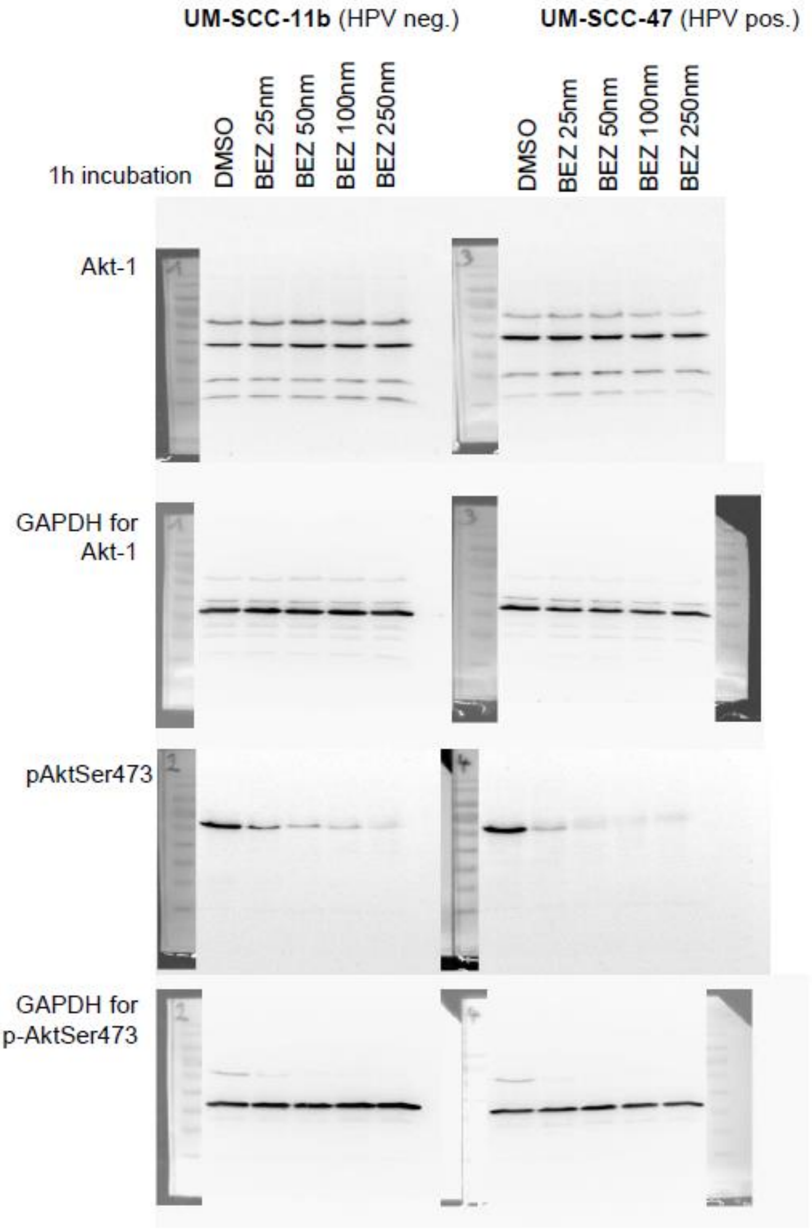

Figure S7. Supplementary data Western Blot raw data corresponding to Figure 1C.

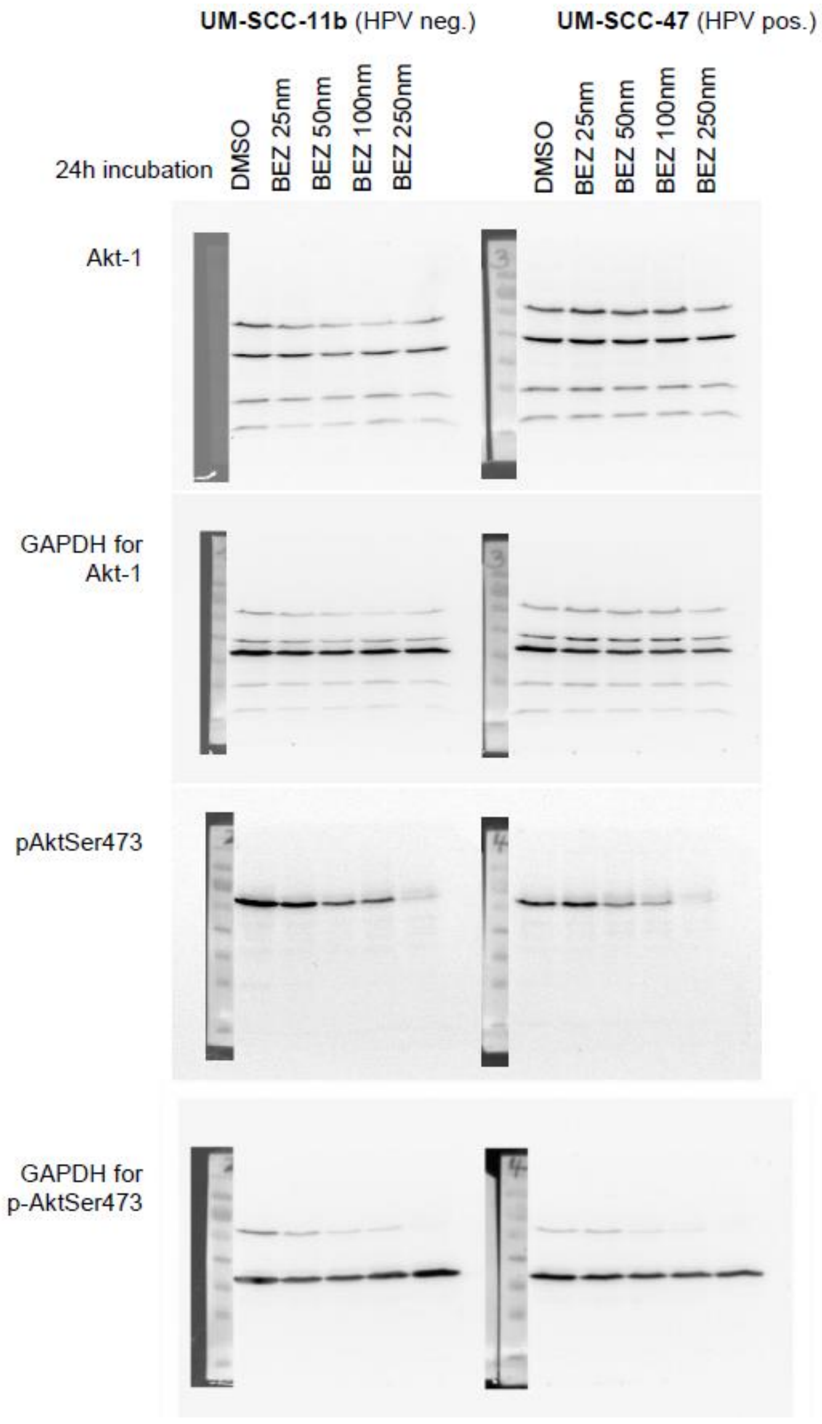

Figure S7. Supplementary data Western Blot raw data corresponding to Figure 1C.

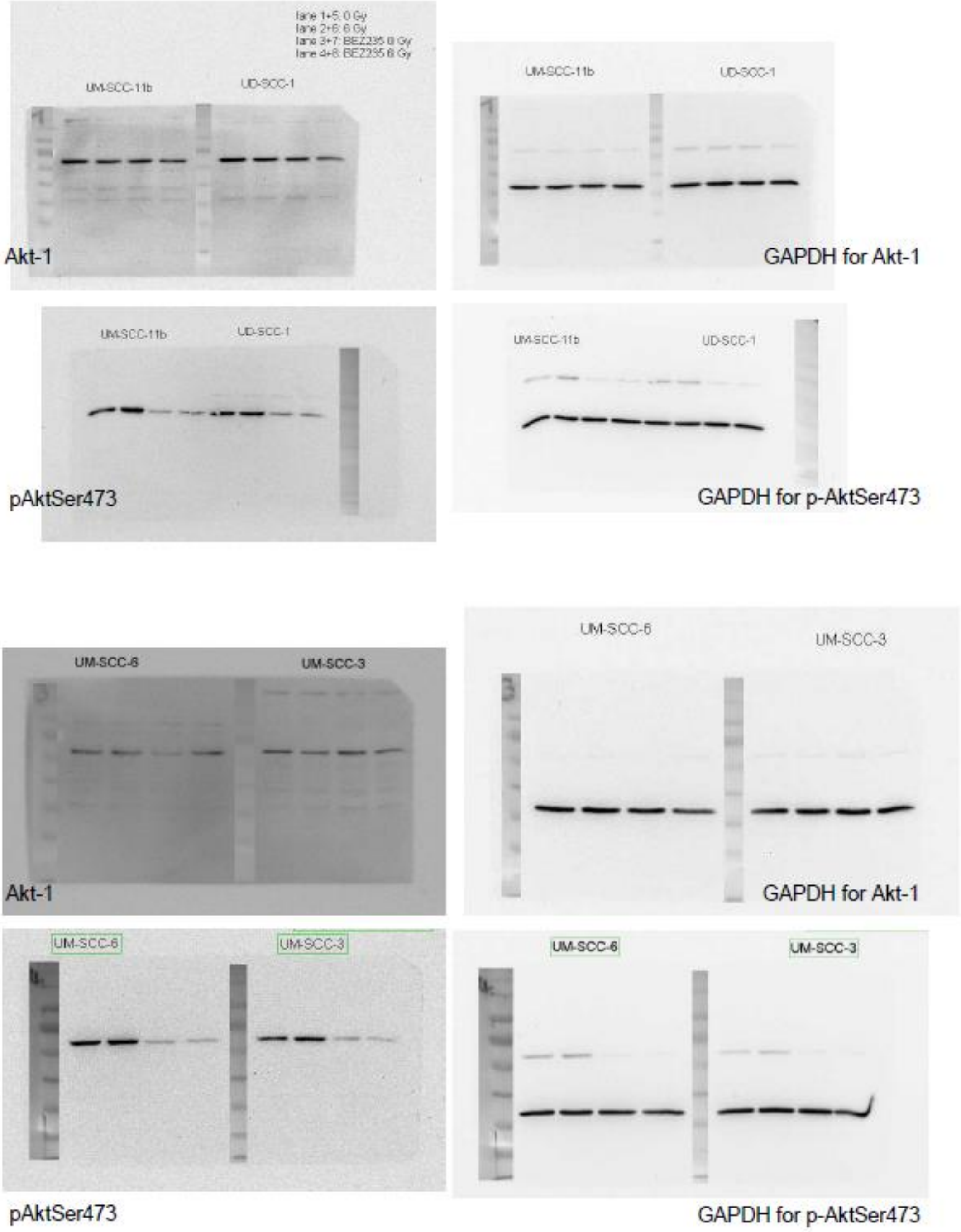

Figure S7. Supplementary data Western Blot raw data corresponding to Figure 1D and E.

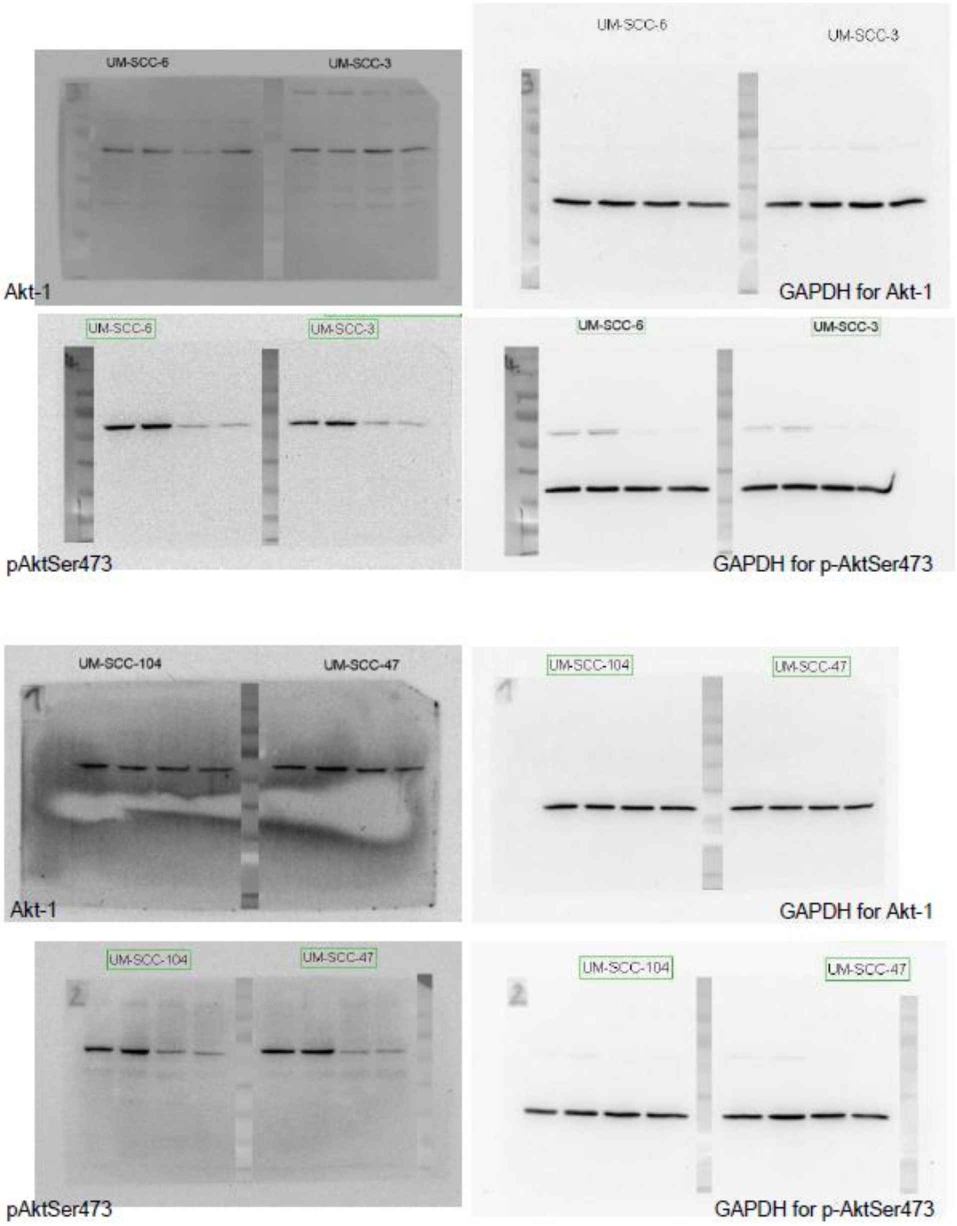

Figure S7. Supplementary data Western Blot raw data corresponding to Figure 1D and E.

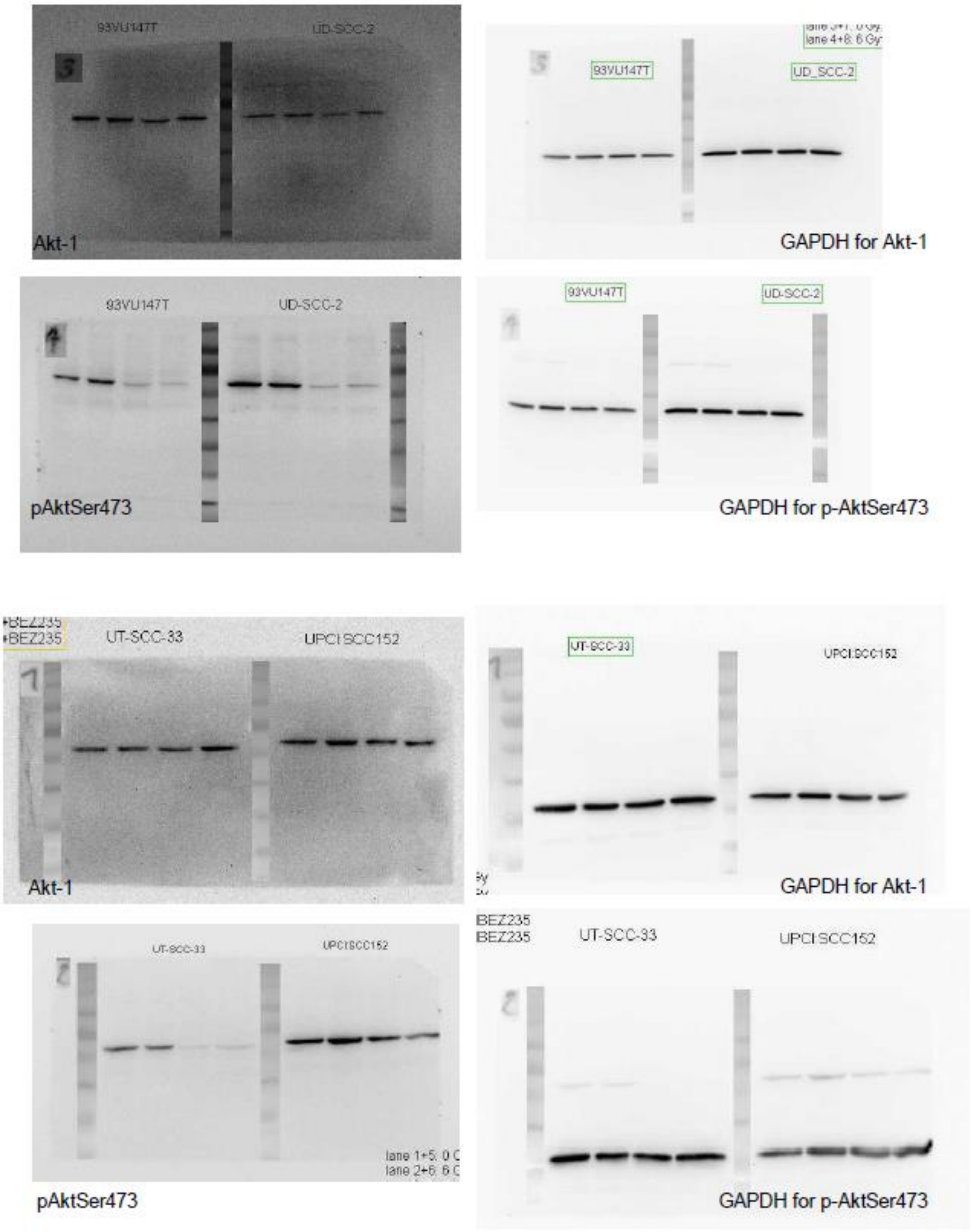

Figure S7. Supplementary data Western Blot raw data corresponding to Figure 1D and E.

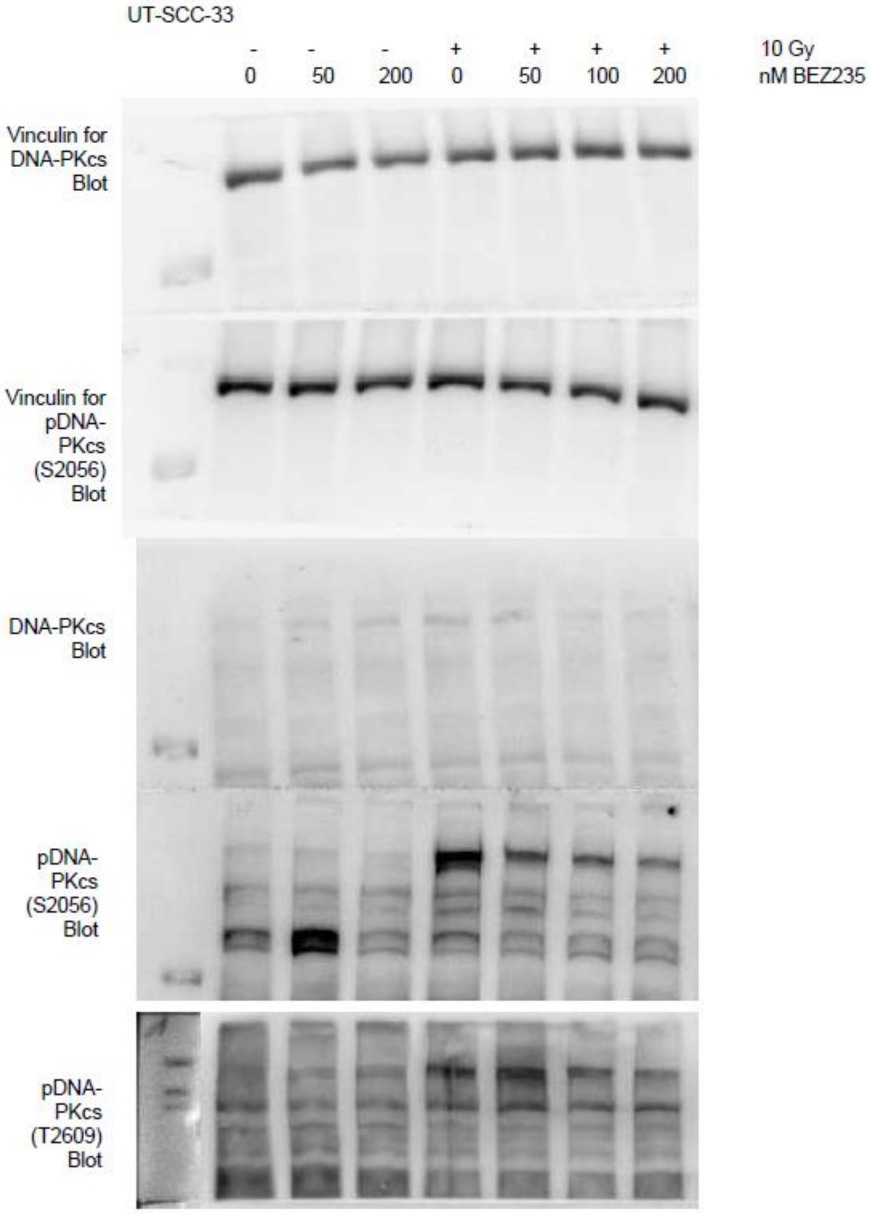

Figure S7. Supplementary data Western Blot raw data corresponding to Figure 4C.

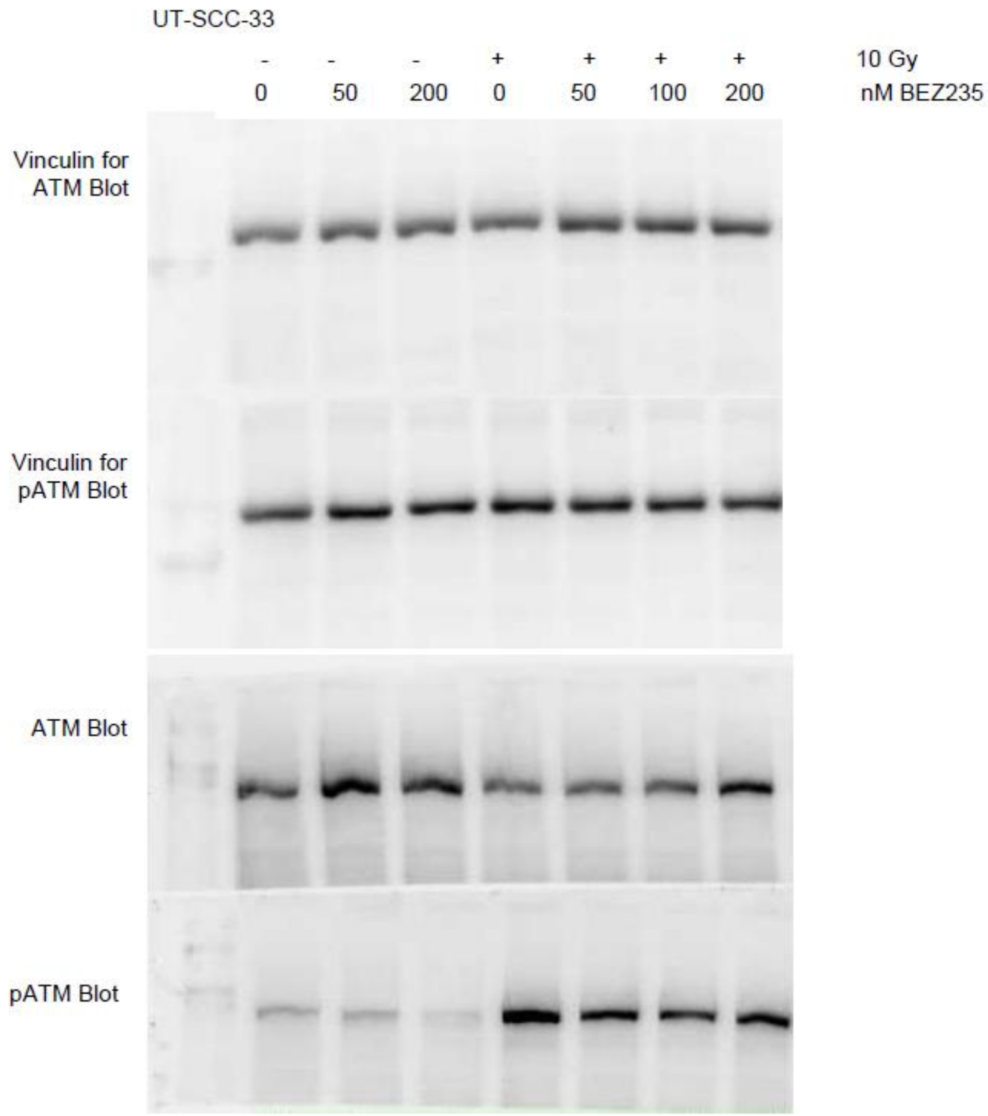

Figure S7. Supplementary data Western Blot raw data corresponding to Figure 4E.

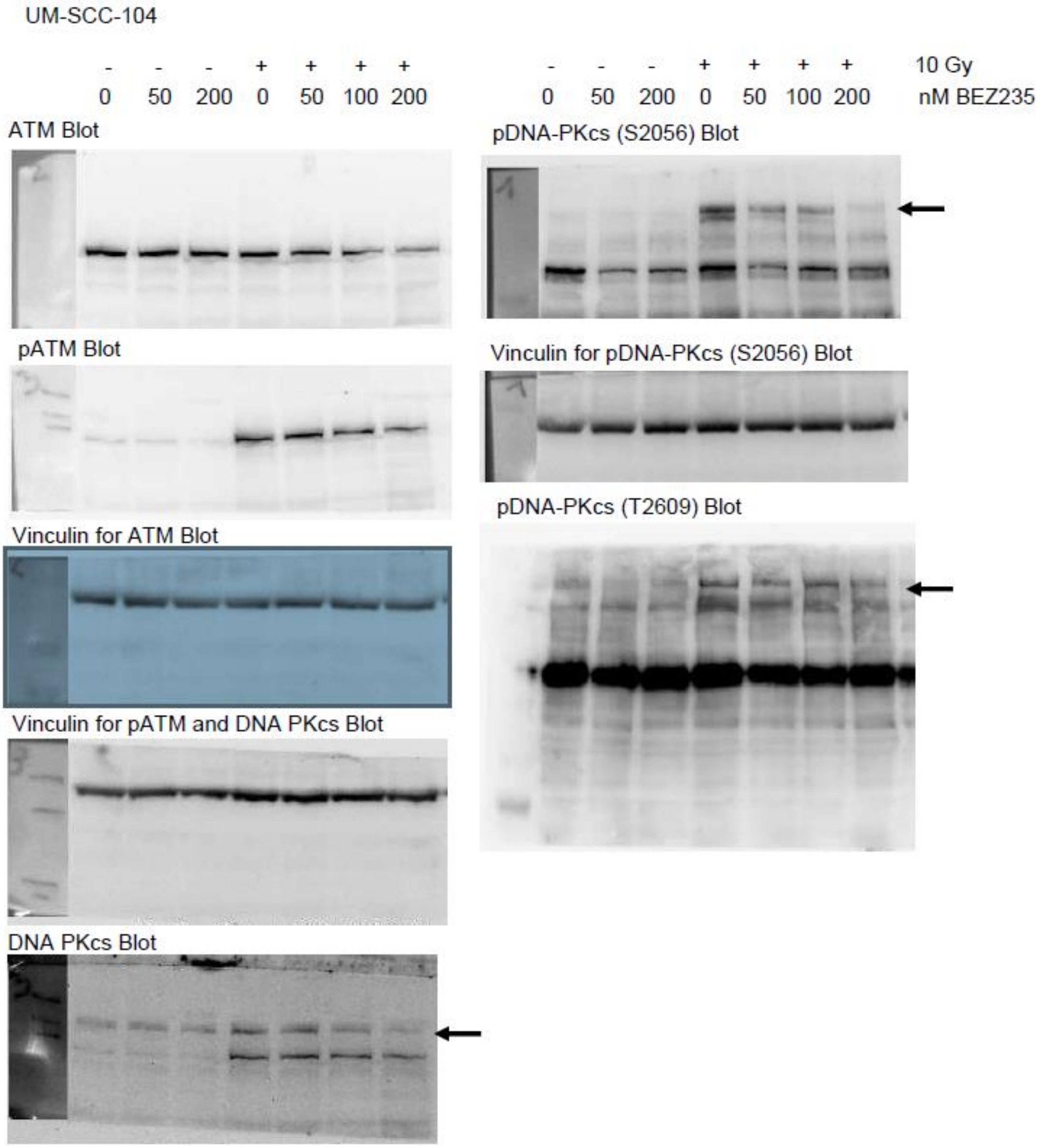

Figure S7. Supplementary data Western Blot raw data corresponding to Figure 4C.
